# Development of key performance indicators to evaluate centralized intake for patients with osteoarthritis and rheumatoid arthritis

**DOI:** 10.1186/s13075-015-0843-7

**Published:** 2015-11-14

**Authors:** Claire E. Barber, Jatin N. Patel, Linda Woodhouse, Christopher Smith, Stephen Weiss, Joanne Homik, Sharon LeClercq, Dianne Mosher, Tanya Christiansen, Jane Squire Howden, Tracy Wasylak, James Greenwood-Lee, Andrea Emrick, Esther Suter, Barb Kathol, Dmitry Khodyakov, Sean Grant, Denise Campbell-Scherer, Leah Phillips, Jennifer Hendricks, Deborah A. Marshall

**Affiliations:** Division of Rheumatology, Department of Medicine, University of Calgary, HRIC Room 3AA20, 3280 Hospital Drive NW, Calgary, AB T2N 4Z6 Canada; Alberta Bone and Joint Health Institute, Calgary, AB Canada; Faculty of Rehabilitation Medicine, University of Alberta, Edmonton, AB Canada; Arthritis Working Group, Bone and Joint Health Strategic Clinical Network, Calgary and Edmonton, AB Canada; Division of Rheumatology, University of Alberta, Edmonton, AB Canada; Alberta Hip and Knee Clinic, Calgary, AB Canada; Edmonton Musculoskeletal Centre, Edmonton, AB Canada; Hip and Knee Working Group, Bone and Joint Health Strategic Clinical Network, Calgary and Edmonton, AB Canada; Strategic Clinical Networks, Alberta Health Services, Calgary, AB Canada; Department of Community Health Sciences, University of Calgary, Calgary, AB Canada; Workforce Research and Evaluation, Alberta Health Services, Calgary, AB Canada; Foothills Medical Centre, Alberta Health Services, Calgary, AB Canada; The RAND Corporation, Santa Monica, CA USA; Department of Family Medicine, University of Alberta, Edmonton, AB Canada

**Keywords:** Osteoarthritis, Rheumatoid arthritis, Health services research

## Abstract

**Introduction:**

Centralized intake is integral to healthcare systems to support timely access to appropriate health services. The aim of this study was to develop key performance indicators (KPIs) to evaluate centralized intake systems for patients with osteoarthritis (OA) and rheumatoid arthritis (RA).

**Methods:**

Phase 1 involved stakeholder meetings including healthcare providers, managers, researchers and patients to obtain input on candidate KPIs, aligned along six quality dimensions: appropriateness, accessibility, acceptability, efficiency, effectiveness, and safety. Phase 2 involved literature reviews to ensure KPIs were based on best practices and harmonized with existing measures. Phase 3 involved a three-round, online modified Delphi panel to finalize the KPIs. The panel consisted of two rounds of rating and a round of online and in-person discussions. KPIs rated as valid and important (≥7 on a 9-point Likert scale) were included in the final set.

**Results:**

Twenty-five KPIs identified and substantiated during Phases 1 and 2 were submitted to 27 panellists including healthcare providers, managers, researchers, and patients in Phase 3. After the in-person meeting, three KPIs were removed and six were suggested. The final set includes 9 OA KPIs, 10 RA KPIs and 9 relating to centralized intake processes for both conditions. All 28 KPIs were rated as valid and important.

**Conclusions:**

Arthritis stakeholders have proposed 28 KPIs that should be used in quality improvement efforts when evaluating centralized intake for OA and RA. The KPIs measure five of the six dimensions of quality and are relevant to patients, practitioners and health systems.

**Electronic supplementary material:**

The online version of this article (doi:10.1186/s13075-015-0843-7) contains supplementary material, which is available to authorized users.

## Introduction

Arthritis is the leading cause of physical disability in Canada and the burden of arthritis, including osteoarthritis (OA) and rheumatoid arthritis (RA), is expected to increase over the next 30 years [1]. Timely diagnosis and appropriate treatment are associated with better outcomes for patients with arthritis. Patients with OA report less pain and improved quality of life and function following timely joint arthroplasties [2–4]. In RA, it is well established that early, targeted treatment with disease-modifying antirheumatic drugs (DMARDs) is associated with improved outcomes [5–7], which is a central component in evidence-based RA guidelines [8–11].

Unfortunately, many patients with OA and RA in Canada experience delays in access to care and treatment. The Canadian Institute for Health Information reports that the proportion of patients meeting wait time benchmarks for elective procedures (including hip and knee replacement) have remained largely unchanged over the last 3 years, and that many patients are not receiving care within recommended benchmarks [12]. In RA, national wait time data are lacking [13]; however, provincial studies suggest that access to rheumatologic care may be problematic because of workforce shortages [14–16].

The reasons for delays in care for appropriate treatment of patients with OA and RA are complex and include a mismatch between supply and demand for specialist care in many regions [14–17]. Furthermore, inefficiencies at the level of referral and triage can lead to delays in care [18]. Effective referral and intake management of this patient population is needed to ensure that the needs of patients are addressed in a timely, organized, transparent and consistent manner.

Centralized intake is a system that facilitates getting the right patients to the right providers at the right time by pooling patients into a single queue, assessing the nature and the urgency of referral, and prioritizing access to care based on the assessment of the referral [19, 20]. Centralized intake systems that incorporate these elements of single-entry models and wait list management are key components to wait time reduction strategies [12]. These strategies have been shown to reduce wait times for specialty care and to improve the effective use of healthcare services [20–22].

The objective of the present work was to develop key performance indicators (KPIs) for use in evaluating centralized intake systems for arthritis care, starting with OA and RA. For the purposes of this work, OA KPIs were focused on those patients with moderate to severe OA who required either surgical (total hip or knee arthroplasty) or nonsurgical management (requiring specialist consultation). The KPIs will be used to measure system improvements in the following dimensions of quality taken from the provincial quality framework [23]:

- *Appropriateness*: whether services are delivered according to best practices and relevant to user needs
- *Accessibility*: whether services are delivered in a timely manner
- *Acceptability*: whether services are responsive to user expectations and preferences
- *Effectiveness*: whether services are based on knowledge to achieve the best outcomes
- *Efficiency*: whether services are optimally used

## Methods

**Fig. 1:**
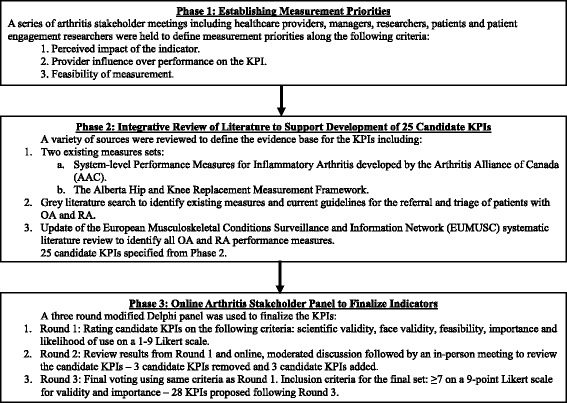
Key performance indicator (KPI) development process. *OA* osteoarthritis, *RA* rheumatoid arthritis

The present work is part of a study to improve access to appropriate and effective arthritis care through collaboration with arthritis stakeholders. The KPIs for centralized intake of arthritis care were developed over three phases, as shown in Fig. 1 and described below.

### Phase 1: Establishing measurement priorities

A series of arthritis stakeholder meetings were convened to define measurement priorities. Throughout this process, the following stakeholder groups were represented: healthcare providers, managers, researchers, patients and patient engagement researchers. The following criteria were used to prioritize KPI development:

1. *Perceived impact*: Are there identifiable health benefits to patients who receive care, to care providers, and to the health system?
2. *Provider influence over performance on the KPI*: Is this factor under the control of a care provider, or can it lead to changes in the healthcare system?
3. *Feasibility of measurement*: Is the required information to measure performance available from data sources (e.g., electronic medical records or administrative data sets)?

### Phase 2: Integrative review of the literature to support candidate key performance indicators

An integrative review of the literature [24, 25] was conducted to ensure that the 25 candidate KPIs were based on evidence and/or best practices and that they were harmonized with any existing published performance measures. The search strategy is shown in Additional file 1.

Briefly, the following sources were used to inform and support KPI development:

1. KPIs pertaining to RA were harmonized with the Arthritis Alliance of Canada (AAC) system-level performance measures for inflammatory arthritis (IA) [26].
2. Existing measures from the Alberta Hip and Knee Replacement Measurement Framework for measuring quality of care for hip and knee arthroplasty [27–29] were considered for inclusion in the harmonized set.
3. A grey literature search was conducted to identify existing measures and current guidelines for the referral and triage of patients with OA and RA by searching the websites of 33 arthritis organizations in North America, Europe, the United Kingdom and Australia.
4. We updated a recent systematic review of the literature conducted by the European Musculoskeletal Conditions Surveillance and Information Network [30] in two literature databases (MEDLINE and Embase) to identify all existing performance measures for OA and RA.

### Phase 3: Online arthritis stakeholder panel to finalize indicators

To finalize the KPIs, a modification of the RAND-UCLA Appropriateness Method [31] was used during a three-round, online, modified Delphi procedure using an online platform called ExpertLens [32, 33].

#### Panel composition and recruitment

Twenty-eight panellists were invited to take part in Phase 3 of KPI development. All members of the panel were from Alberta, and they included healthcare providers (including rheumatologists, orthopaedic surgeons, primary care physicians and triage personnel), managers (including clinic managers and health administrators), and researchers and patients, including some who are trained in patient and community engagement research. Panellists were recruited to ensure representation from the major arthritis care centres as well as from large primary care networks and patient groups from across Alberta. No honoraria or incentives were offered for participation. The University of Calgary Conjoint Health Research Ethics Board approved this study (REB13-0822_MOD5), and the RAND Corporation’s Human Subjects Protection Committee exempted the study from review (study identifier 2015-0005). The participants in this study provided consent to participate and for us to publish the study findings.

#### Panel protocol

In round 1, panellists rated the candidate KPIs anonymously using the online ExpertLens platform. In round 2, panellists reviewed the results of the round 1 voting and were given the opportunity to participate anonymously in an asynchronous online discussion about the candidate KPIs. Following this, an in-person meeting was held to review votes and comments on each KPI from rounds 1 and 2. By consensus, some KPIs were removed and others were modified or added to better reflect the measurement priorities. Minor modifications to wording or specification of the remaining KPIs were made. In round 3, panellists voted again on the modified KPI set using the same questions asked in round 1.

After reviewing a background document that described the KPI development process, rationale for measurement, and supporting information, the panellists rated each KPI based on the following criteria on a 9-point Likert-type rating scale:

1. *Scientific validity*: How strong is either the scientific evidence or professional consensus supporting this indicator?
2. *Face validity*: How likely is it that better performance on the proposed indicator reflects a higher-quality health system?
3. *Feasibility*: How likely is it that the information required to report on this indicator will be available in your health system?
4. *Importance*: How important is it to measure and report on this indicator when evaluating centralized intake for arthritis care?
5. *Likelihood of use*: How likely is it that you would use, or encourage the use of, this indicator for quality improvement in your centre?

For the KPIs that were harmonized with the AAC performance measures, validity was already established using a similar process [26]; therefore, participants were asked to answer only questions 3, 4 and 5.

#### Analysis of panellist responses

To be included in the final set, the KPIs had to be rated as highly scientifically valid and of high importance (questions 1 and 4, median ratings ≥7 on a 9-point scale with no disagreement). Disagreement was calculated according to the RAND/UCLA Appropriateness Method handbook [31]. Disagreement exists when the interpercentile range (IPR) (difference between the 30th and 70th percentiles) is larger than the Interpercentile Range Adjusted for Symmetry (IPRAS), which was calculated using the formulae: IPRAS = 2.35 + [asymmetry index (AI) × 1.5] [31], where the AI is the absolute difference between 5 and the central point of the IPR (IPRCP) [31, 34].

Similarly, to include the KPIs that were harmonized with the AAC measures in the final set, there had to be agreement on the importance and likelihood of use (median ratings ≥7 on a 9-point scale).

The feasibility of measuring and reporting on all the identified KPIs will be tested in later studies and may vary for different centres within Alberta. Thus, high feasibility (median ratings ≥7 without disagreement) was not a requirement for inclusion in the final set. However, where there was evidence of panellist uncertainty regarding the feasibility of KPI measurement (median ratings of 4–6 on question 3), KPIs have to be deemed important and highly likely to be used as indicators for quality improvement (median ratings ≥7 on a 9-point scale with no disagreement) to be included in the final set.

## Results

### Establishing measurement priorities (Phase 1)

An overview of the process used to establish measurement priorities is shown in Fig. 1. In summary, the major strategic decisions made regarding the scope of the measures included the following:

1. The KPIs were selected to capture important steps along the continuum of care between referral submission to diagnosis and treatment (see Fig. 2). The stakeholders acknowledged that guidelines and high-quality evidence might be lacking for measurement of some of the candidate KPIs (e.g., measuring time from receipt of referral to completion, or measuring patient or provider experience with centralized intake). Therefore, professional consensus was deemed an acceptable level of evidence for development and inclusion of such candidate KPIs.

**Fig. 2:**
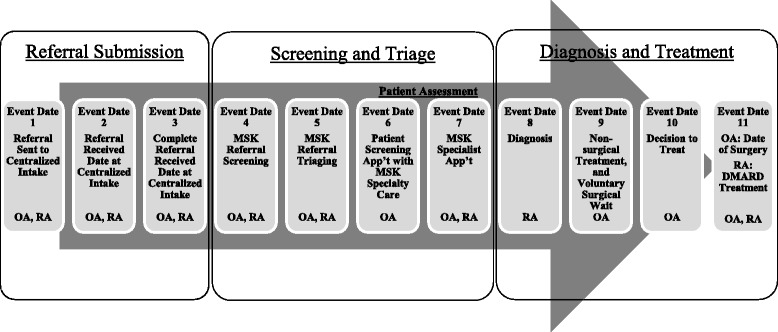
Example of a patient flow diagram for patients with osteoarthritis (OA) and patients with rheumatoid arthritis (RA) who are referred to centralized intake. *Musculoskeletal (MSK) referral screening*: clerical review of incoming referrals to quickly identify if referrals are complete, and which patients should be referred for a patient assessment to formally evaluate their MSK care needs. *MSK referral triaging*: review of screened referrals to establish urgency and prioritize patients for patient assessment based on disease and severity of symptoms. *MSK Specialty Care*: secondary care providers with MSK expertise, including specialists such as orthopaedic surgeons, rheumatologists, physiotherapists and nurses. *MSK Specialist Care*: subset of specialty care providers (i.e., specialized physicians, orthopaedic surgeons and rheumatologists). *DMARD* biologic and nonbiologic disease-modifying antirheumatic drug
2. Although the stakeholders anticipate that, by improving access to care and treatment, patient outcomes will improve, measurement of long-term patient outcomes was felt to be outside the scope of the present project.
3. Although all dimensions of quality of care were considered, safety indicators (such as drug monitoring) would not be part of the final KPI set. This decision was made because the role of centralized intake is to facilitate access to the most appropriate provider in a timely fashion, but it does not necessarily include treatment of patients and subsequent safety monitoring.

As a result of Phase 1, 25 KPIs were suggested for further development based on the criteria for prioritization and the strategic decisions listed above.

### Integrative literature review (Phase 2)

Thirteen KPIs were harmonized with existing measures (see Table 1 for measure sources). The remainder of the KPIs were supported by guidelines or evidence supporting best practices for centralized intake found in our integrative review (full review results available upon request).

**Table 1:** Key performance indicators for musculoskeletal centralized intake

Key performance indicator	Dimension of quality of care	Derived from existing measure	Included or removed after round 2
1. Time from osteoarthritis referral receipt to referral completion for initially incomplete referrals	Accessibility, efficiency	New KPI	Included
2. Time from rheumatoid arthritis referral receipt to referral completion for initially incomplete referrals	Accessibility, efficiency	New KPI	Included
3. Percentage of osteoarthritis referrals received with complete information	Efficiency	Hip and Knee Replacement Measurement Framework^a,b^	Included
4. Percentage of rheumatoid arthritis referrals received with complete information	Efficiency	New KPI	Included
5. Time from receipt of complete osteoarthritis referral to musculoskeletal appointment	Accessibility	Hip and Knee Replacement Measurement Framework^a,b^	Included
6. Waiting times for rheumatologist consultation for patients with new-onset rheumatoid arthritis	Accessibility	AAC Performance measures for IA^c^	Included
7. Time to disease-modifying antirheumatic drug therapy for patients with new-onset rheumatoid arthritis	Accessibility, effectiveness	AAC Performance measures for IA^c^	Included
8. Percentage of patients with new-onset rheumatoid arthritis with at least one visit to a rheumatologist in the first year of diagnosis	Accessibility	AAC Performance measures for IA^c^	Included
9. Orthopaedic surgeons per 100,000 population	Accessibility	COA^d,e^, AAOS^f^, ACREU^e,g^	Removed
10. Rheumatologists per 100,000 population	Accessibility	AAC Performance measures for IA^c^	Included
11. Percentage of patients that receive information regarding resources and tools available for management while waiting for first musculoskeletal specialty contact	Appropriateness	Hip and Knee Replacement Measurement Framework^a,b^	Included
12. Percentage of osteoarthritis referrals scored using Western Canada Waiting List priority referral criteria^h^	Appropriateness	New KPI	Included
13. Distribution of osteoarthritis referrals in each urgency category (as scored using the Western Canada Waiting List referral tool)	Appropriateness	New KPI	Included
14. Percentage of osteoarthritis referrals triaged as highest urgency based on high Western Canada Waiting List priority criteria scores seen within Wait Time Alliance benchmarks	Appropriateness	New KPI	Included
15. Percentage of rheumatoid arthritis referrals assessed using a priority tool	Appropriateness	New KPI	Removed
16. Percentage of rheumatoid arthritis referrals categorized as early rheumatoid arthritis	Appropriateness	New KPI	Removed
17. Waiting times for patients with established rheumatoid arthritis	Accessibility	New KPI	Included
18. Percentage of rheumatoid arthritis patients treated with a disease-modifying antirheumatic drug during the measurement year	Effectiveness, accessibility	AAC Performance measures for IA^c^	Included
19. Percentage of referrals rejected or redirected when received at centralized intake	Appropriateness	New KPI	Included
20. Percentage of musculoskeletal appointments completed as scheduled	Efficiency	Hip and Knee Replacement Measurement Framework^a,b^	Included
21. Percentage of specialist providers participating in centralized intake	Efficiency	New KPI	Included
22. Number of referrals received through centralized intake	Efficiency	New KPI	Included
23. Patient experience with centralized intake	Acceptability	AHRQ^i^, NHS^j^, and Ministry of Health and Long-Term Care (Ontario)^k^	Included
24. Referring clinician experience with centralized intake	Acceptability	The Ministry of Health and Long-Term Care (Ontario)^k^	Included
25. Musculoskeletal specialty care provider experience with centralized intake	Acceptability	The Ministry of Health and Long-Term Care (Ontario)^k^	Included
26. Ratio of patient flow to estimated clinic capacity of osteoarthritis teams participating in centralized intake	Efficiency, accessibility	Developed during round 2	N/A
27. Operating room time for arthroplasty surgeons in Alberta	Accessibility	Developed during round 2	N/A
28. Ratio of patient flow to clinic capacity of rheumatoid arthritis teams participating in centralized intake	Efficiency, accessibility	Developed during round 2	N/A
29. Administrative staff and allied health professional experience with centralized intake	Acceptability	Developed during round 2	N/A
30. Agreement of centralized intake suspected diagnosis of severe osteoarthritis cases (e.g., patients who are candidates for hip or knee joint replacements) versus confirmed diagnosis of severe osteoarthritis	Appropriateness, effectiveness	Developed during round 2	N/A
31. Agreement of centralized intake suspected diagnosis versus confirmed diagnosis for rheumatoid arthritis	Appropriateness, effectiveness	Developed during round 2	N/A
		Total number of candidate KPIs before round 2: 25	Total number of candidate KPIs after round 2: 28

*AAC* Arthritis Alliance of Canada, *IA* Inflammatory Arthritis, *AAOS* American Academy of Orthopaedic Surgeons, *ACREU* Arthritis Community Research and Evaluation Unit, *AHRQ* Agency for Healthcare Research and Quality, *COA*, Canadian Orthopaedic Association, *NHS*, National Health Service

^a^Frank et al. [27]

^b^Marshall et al. [39]

^c^Barber et al. [26]

^d^Rumble and Kreder [40]

^e^Badley et al. [17]

^f^Natividad [41]

^g^Canizares et al. [42]

^h^The Western Canada Waiting List (WCWL) Project has developed and validated a hip and knee replacement priority criteria tool to assess clinical urgency for hip and knee joint replacements in a standardized and reliable manner [43]. The hip and knee replacement priority criteria tool is a clinician-scored tool consisting of seven items: (1) pain on motion, (2) pain at rest, (3) ability to walk, (4) other functional limitations, (5) abnormal findings, (6) potential for progression of disease and (7) ability to work, give care to dependents and live independently. The urgency is determined through a point count scoring system and could be used to structure and manage waiting lists for hip and knee joint replacements [44]

^i^Camacho et al. [45]

^j^Clinical Indicators Team [46]

^k^Deloitte & Touche LLP and affiliated entities [47]

### Online modified Delphi procedure to finalize KPIs (Phase 3)

The candidate set submitted for panel evaluation is shown in Table 1. It included KPIs to address performance of centralized intake in the following categories: seven OA-specific KPIs, ten RA-specific KPIs and eight KPIs that apply to centralized intake systems for both conditions.

### Response rates and participant demographics

Twenty-four (85.7 %) of the twenty-eight invitees participated in round 1 of the modified Delphi process, and twenty-three (82.1 %) participated in round 3. Four participants participated in round 1 but not in round 3, and three participants participated in round 3 but not in round 1. In total, 27 (96.4 %) of the 28 invitees participated in at least one round. During round 2, 12 participants (42.9 %) contributed to the online platform discussion and 19 (67.9 %) contributed during the in-person meeting. One of the participants who participated in the online discussion was not present at the meeting, so the total participation rate during round 2 was 20 (71.4 %).

Twenty-six participants (93 %) provided basic demographic information: ten (39 %) were physicians (four primary care physicians, four rheumatologists and two orthopaedic surgeons); three were patient representatives, including some patient engagement researchers (12 %); one was a triage nurse (4 %); four were researchers (15 %); four were healthcare managers (15 %); and four described themselves as ‘other’ (15 %). Twenty-five participants provided information about their geographic area. Eleven were from Calgary (44 %), nine were from Edmonton (36 %) and five were from other regions in Alberta (20 %).

### Results from online provincial panel to finalize KPIs (Phase 3, round 2)

During the round 2 in-person meeting, the results from round 1 and the online discussion part of round 2 were reviewed (data available upon request). Based on discussion of the results of these previous rounds, some modifications to the KPI set were made (Table 1). Three of the original KPIs were removed from the final round of voting by consensus. KPI 9 (orthopaedic surgeons per 100,000 population) was excluded because panellists felt there were better ways of capturing surgical capacity; they suggested alternate KPIs (see description below for KPIs 26 and 27). KPI 15 (percentage of RA referrals assessed using a priority tool) was excluded due to concerns of overly burdening the referring physicians. KPI 16 (percentage of RA referrals categorized as early RA) was excluded, as it was deemed not sufficiently important and was superseded by the other KPIs relating to wait times to receive care.

The panellists also recommended a number of new KPIs that more effectively captured the measurement priorities. KPI 26 (ratio of patient flow to estimated clinic capacity of OA teams participating in centralized intake) and KPI 27 (operating room time for arthroplasty surgeons in Alberta) were added to more adequately reflect the capacity for complex OA care. Together, they replaced the number of orthopaedic surgeons per 100,000 population (KPI 9). The analogous RA KPI—rheumatologists per 100,000 population (KPI 10)—was still included because of greater concern regarding rheumatologist capacity to provide high-quality RA care. An additional RA indicator, KPI 28 (ratio of patient flow to clinic capacity of RA teams participating in centralized intake) was added to capture clinic capacity.

To capture acceptability of centralized intake, the original set of KPIs included three indicators (KPIs 23–25) that measured the patient, referring clinician and arthritis specialty provider’s experience with the centralized intake system. The panellists felt that the administrative staff and allied health professionals should also be included in a separate indicator and thus suggested KPI 29 (administrative staff and allied health professional experience with centralized intake).

The panellists related that an important function of centralized intake is ensuring that patients are appropriately triaged based on the suspected diagnosis and the information provided on the referral form. To assess this, KPIs related to the degree of agreement between the centralized intake suspected diagnosis and the specialist’s final diagnosis were suggested by the panellists to be a critical component for evaluating the effectiveness of triage. Thus, two new indicators were added to evaluate the diagnostic agreement, one each for OA (KPI 30) and RA (KPI 31).

### Final round 3 voting

A total of 28 KPIs were submitted for final voting in round 3. Ten RA-specific KPIs (Table 2), nine OA-specific KPIs (Table 3) and nine KPIs applied to centralized intake systems for both conditions (Table 4). All ten RA KPIs were rated as valid, feasible and important, with a high perceived likelihood of use (median ratings ≥7 on a 9-point scale) and no disagreement according to the IPR > IPRAS rule. Similarly, seven of the nine KPIs for OA were rated highly in all domains, with two exceptions. KPI 12 (percentage of OA referrals scored using Western Canada Waiting List (WCWL) [35] priority referral criteria) had median scores of 6 for face validity and feasibility, and KPI 13 (distribution of OA referrals in each urgency category scored using the WCWL referral tool) had a median score of 6 in feasibility. Of the nine KPIs that applied to centralized intake systems in general, there was uncertainty regarding the feasibility of capturing three: KPI 11 (percentage of patients who receive information regarding resources and tools available for management while waiting for first musculoskeletal specialty contact), KPI 24 (referring clinician’s experience with centralized intake) and KPI 25 (musculoskeletal specialty care provider experience with centralized intake). All other KPIs had median scores ≥7 for validity, importance and likelihood of use, with no disagreement.

**Table 2:** Final round 3 voting on ten rheumatoid arthritis–specific key performance indicators for centralized intake

	Median (range) and percentage of participants voting ≥7 on 1–9 scale for each domain (n = 23 unless otherwise specified)				
	Scientific validity	Face validity	Feasibility: information availability	Importance	Likelihood of use
KPI 2: Time from RA referral receipt to referral completion for initially incomplete referrals	7 (5–8), 61 %	7 (6–8), 74 %	8 (6–8), n = 22 (64 %)	8 (7–8), 78 %	7 (6–8), 65 %
KPI 4: Percentage of RA referrals received with complete information	7 (7–8), 78 %	8 (7–8), 83 %	7 (6–8), n = 22 (59 %)	8 (7–8), n = 22 (82 %)	7 (6–8), n = 22 (68 %)
KPI 6: Waiting times for rheumatologist consultation for patients with new-onset rheumatoid arthritis	N/A^a^	N/A^a^	8 (8–9), n = 21 (95 %)	9 (8–9), n = 22 (100 %)	9 (9–9), n = 22 (100 %)
KPI 7: Time to disease-modifying antirheumatic drug therapy for patients with new-onset RA	N/A^a^	N/A^a^	7 (6–8), n = 22 (68 %)	9 (8–9), 91 %	9 (8–9), n = 22 (100 %)
KPI 8: Percentage of patients with new-onset RA with at least one visit to a rheumatologist in the first year of diagnosis	N/A^a^	N/A^a^	7 (6–8), n = 21 (57 %)	8 (7–9), 78 %	8 (7–8), 83 %
KPI 10: Rheumatologists per 100,000 population	N/A^a^	N/A^a^	7 (6–8), n = 21 (62 %)	7 (6–7), n = 22 (59 %)	7 (5–7), n = 21 (52 %)
KPI 17: Waiting times for patients with established RA	8 (8–9), 96 %	9 (8–9), 96 %	8 (7–8), n = 22 (77 %)	8 (8–9), 91 %	8 (8–9), 96 %
KPI 18: Percentage of rheumatoid arthritis patients treated with a disease-modifying antirheumatic drug during the measurement year	N/A^a^	N/A^a^	8 (6–9), 70 %	8 (8–9), 83 %	9 (8–9), 83 %
KPI 28: Ratio of patient flow to clinic capacity of RA teams participating in centralized intake	7 (6–8), n = 22 (73 %)	8 (7–8), n = 22 (82 %)	7 (5–8), n = 22 (55 %)	7 (7–8), 83 %	8 (7–9), n = 22 (86 %)
KPI 31: Agreement of centralized intake suspected diagnosis versus confirmed diagnosis of RA	8 (6–8), n = 21 (71 %)	8 (6–8), 74 %	7 (5–8), n = 22 (55 %)	8 (7–9), n = 22 (86 %)	8 (7–9), n = 21 (76 %)

*RA* rheumatoid arthritis

^a^Key performance indicators (KPIs) 6, 7, 8, 10 and 18 were harmonized with the Arthritis Alliance of Canada performance measure set for inflammatory arthritis which used a similar process for development and scientific validity and face validity were not examined again in the present study

**Table 3:** Final round 3 voting on nine osteoarthritis-specific key performance indicators for centralized intake

	Median (range) and percentage of participants voting ≥7 on 1–9 scale for each domain (n = 23 unless otherwise specified)				
	Scientific validity	Face validity	Feasibility: information availability	Importance	Likelihood of use
KPI 1: Time from OA referral receipt to referral completion for initially incomplete referrals	7 (5–8), 65 %	7 (6–8), 74 %	8 (6–8), n = 22 (68 %)	8 (7–8), 78 %	7 (6–8), 74 %
KPI 3: Percentage of OA referrals received with complete information	7 (6–8), 65 %	8 (7–8), 83 %	7 (6–8), n = 22 (73 %)	7 (7–8), 83 %	7 (6–8), n = 22 (64 %)
KPI 5: Time from receipt of complete OA referral to musculoskeletal appointment	8 (8–9), 96 %	9 (8–9), 100 %	8 (7–9), n = 22 (86 %)	9 (8–9), n = 22 (100 %)	9 (8–9), n = 22 (95 %)
KPI 12: Percentage of OA referrals scored using Western Canada Waiting List priority referral criteria	7 (6–7), n = 22 (73 %)	6 (6–8), n = 22 (45 %)	6 (5–7), n = 22 (45 %)	7 (6–7), n = 22 (64 %)	7 (6–7), n = 22 (59 %)
KPI 13: Distribution of OA referrals in each urgency category (as scored using the Western Canada Waiting List referral tool)	7 (7–8), 83 %	7 (6–8), 65 %	6 (6–7), n = 22 (50 %)	7 (6–8), 74 %	7 (6–8), 61 %
KPI 14: Percentage of OA referrals triaged as highest urgency based on high Western Canada Waiting List priority criteria scores seen within Wait Time Alliance benchmarks	8 (7–8), 96 %	8 (7–8), 91 %	7 (6–7), n = 22 (59 %)	7 (7–8), n = 21 (95 %)	8 (7–8), 87 %
KPI 26: Ratio of patient flow to estimated clinic capacity of OA teams participating in centralized intake	7 (6–7), n = 22 (64 %)	8 (7–8), 83 %	7 (5–7), n = 22 (55 %)	8 (7–9), n = 22 (82 %)	8 (7–9), n = 22 (82 %)
KPI 27: Operating room time for arthroplasty surgeons in Alberta	7 (6–8), n = 20 (55 %)	7 (7–8), n = 22 (77 %)	7 (7–8), n = 20 (75 %)	7 (6–8), n = 21 (67 %)	7 (5–8), n = 21 (67 %)
KPI 30: Agreement of centralized intake suspected diagnosis of severe OA cases (e.g., patients who are candidates for hip or knee joint replacements) versus confirmed diagnosis of severe OA	8 (7–8), n = 21 (81 %)	8 (7–8), n = 21 (90 %)	7 (6–8), n = 19 (63 %)	8 (8–9), n = 21 (95 %)	8 (8–9), n = 19 (89 %)

*KPI* key performance indicator, *OA* osteoarthritis

**Table 4:** Final round 3 voting on nine key performance indicators for centralized intake applicable to rheumatoid arthritis and osteoarthritis

	Median (range) and % of participants voting ≥7 on a 1–9 scale for each domain (n = 23 unless otherwise specified)				
	Scientific validity	Face validity	Feasibility: information availability	Importance	Likelihood of use
KPI 11: Percentage of patients who receive information regarding resources and tools available for management while waiting for first musculoskeletal specialty contact	7 (7–8), 78 %	7 (7–8), 91 %	6 (4–6), n = 21 (24 %)	7 (7–8), n = 22 (86 %)	8 (7–8), n = 22 (77 %)
KPI 19: Percentage of referrals rejected or redirected when received at centralized intake	7 (6–8), 74 %	7 (6–8), 70 %	7 (6–8), n = 21 (67 %)	7 (7–8), 83 %	7 (7–8), n = 22 (77 %)
KPI 20: Percentage of musculoskeletal appointments completed as scheduled	7 (6–8), 65 %	8 (7–8), 87 %	7 (6–8), n = 22 (64 %)	9 (8–9), n = 21 (81 %)	8 (7–9), n = 22 (77 %)
KPI 21: Percentage of specialist providers participating in centralized intake	7 (6–7), 70 %	7 (7–8), 78 %	7 (6–8), n = 22 (73 %)	8 (7–8), 83 %	7 (7–8), 83 %
KPI 22: Number of referrals received through centralized intake	7 (7–8), 78 %	8 (7–8), 87 %	9 (7–9), n = 21 (81 %)	9 (8–9), 100 %	8 (7–9), n = 22 (95 %)
KPI 23: Patient experience with centralized intake	7 (7–8), 87 %	8 (8–9), 87 %	7 (5–8), 65 %	9 (8–9), n = 22 (95 %)	9 (8–9), 87 %
KPI 24: Referring clinician’s experience with centralized intake	7 (7–8), 78 %	8 (7–8), 96 %	5 (5–7), 32 %	9 (8–9), 96 %	9 (7–9), 96 %
KPI 25: Musculoskeletal specialty care provider experience with centralized intake	7 (6–8), 70 %	7 (7–8), 91 %	6 (5–7), n = 22 (45 %)	8 (7–9), 91 %	8 (7–9), 96 %
KPI 29: Administrative staff and allied health professional experience with centralized intake	7 (6–8), n = 22 (73 %)	8 (7–8), 87 %	7 (5–7), n = 22 (59 %)	8 (7–9), 83 %	8 (7–9), n = 21 (76 %)

*KPI* key performance indicator

## Discussion

Through a rigorous process involving arthritis stakeholders from across the Province of Alberta, we developed a set of 28 KPIs for evaluation of centralized intake methods for OA and RA care. The KPIs address five dimensions of the provincial quality framework [23], with the exception of safety, which was deemed outside the scope of this project. Our panellists rated the KPIs as highly valid and important. They also suggested that the indicators were highly likely to be used in the evaluation of centralized intake.

To our knowledge, this is the first set of KPIs specifically designed to assess centralized intake systems for arthritis care. Although developed within the context of Alberta, the KPIs are likely highly relevant to other arthritis and musculoskeletal care settings where centralized intake is feasible. The KPIs may also help to inform improvements in healthcare systems interested in developing centralized intake systems, although such systems may not be possible in all care settings (e.g., single-practice settings). These KPIs may also be of interest to other specialty services.

The KPIs presented herein focus on OA and RA, although it was recognized that any centralized intake system for arthritis care is likely to receive referrals for other types of arthritis. OA and RA were chosen because these are the two most common arthritis conditions in the general population and represent prototypical inflammatory and non-inflammatory arthritis. There is also strong evidence that better access to care and treatment in these conditions leads to improved patient outcomes [2, 3, 5–7, 36, 37].

During the KPI development process, we decided a priori not to exclude KPIs with lower feasibility ratings (median scores of 4–6), reflecting the panellists’ uncertainty about the availability of information. We did not want to exclude potentially important and relevant KPIs for which system changes could be implemented in the future to enable data capture. In total, two OA KPIs related to scoring the WCWL (KPIs 12 and 13), which is a prioritization tool for hip and knee OA surgical consultation [35], were rated as less feasible. During round 2 discussions, it became clear that although the WCWL is included on current triage forms for hip and knee surgical referrals in Alberta, the tool is not empirically scored. It was likely that panellists considered the feasibility of scoring the WCWL in this environment as uncertain because technology changes would be needed to implement scoring of the WCWL. For similar reasons, the face validity of KPI 12 was questioned (percentage of OA referrals scored using the WCWL priority referral criteria). That KPI was retained, however, because panellists indicated that it was sufficiently important and that they were highly likely to use it.

KPI 11 (percentage of patients who receive information regarding resources and tools available for management while waiting for first musculoskeletal specialty contact) also received lower feasibility scores. This is likely because there are few means of tracking which information patients receive while waiting for their appointment (beyond appointment scheduling information), and changes to existing clinical databases and triage processes would be needed to capture this KPI.

Similarly, the KPIs related to the referring clinician or specialty care provider (e.g., rheumatologist or orthopaedic surgeon) experience with centralized intake (KPIs 24 and 25) received lower feasibility ratings, as surveys capturing clinician experience are not routinely done in Alberta. Interestingly, there were higher ratings for KPIs capturing the experience of patients as well as administrative staff and allied health professionals. This may be because there are existing patient surveys already administered routinely in Alberta (especially for patients with OA). Furthermore, it was felt that administrative staff and allied health professionals were more easily surveyed than referring or specialty physicians.

## Conclusion

We developed a set of 28 KPIs for evaluation of centralized intake for patients with OA and RA. The KPIs will be tested further for feasibility using existing data sources (e.g., administrative data and clinical databases) before widespread implementation. Once tested for feasibility, the KPIs will be used to develop and evaluate an optimal centralized intake system for arthritis care for OA and RA. Measuring the impact of changes to a centralized intake system using standardized metrics is critical for ongoing quality assurance and quality improvement in health systems [38], and this work represents a critical first step in optimizing access to healthcare delivery for patients with OA and RA.

## Additional file

Additional file 1:
**Integrative literature review search strategy.** The search strategy used for an integrative review of literature to ensure candidate key performance indicators were based on evidence and/or best practices, and that they were harmonized with any existing published performance measures. (DOCX 20 kb)

## Abbreviations

AAC: Arthritis Alliance of Canada
AAOS: American Academy of Orthopaedic Surgeons
ACREU: Arthritis Community Research and Evaluation Unit
AHRQ: Agency for Healthcare Research & Quality
AI: asymmetry index
COA: Canadian Orthopaedic Association
DMARD: disease-modifying antirheumatic drug
IA: inflammatory arthritis
IPR: interpercentile range
IPRAS: Interpercentile Range Adjusted for Symmetry
KPI: key performance indicator
MSK: musculoskeletal
N/A: not applicable
NHS: National Health Service
OA: osteoarthritis
RA: rheumatoid arthritis
WCWL: Western Canada Waiting List
