# Supplementary material for: Development of key performance indicators to evaluate centralized intake for patients with osteoarthritis and rheumatoid arthritis
Source: Arthritis Res Ther. 2015 Nov 14;17:322. doi: 10.1186/s13075-015-0843-7 (PMC4644283; doi:10.1186/s13075-015-0843-7)
Supplement: Additional file 1: — Integrative literature review search strategy. The search strategy used for an integrative review of literature to ensure candidate key performance indicators were based on evidence and/or best practices, and that they were harmonized with any existing published performance measures. (DOCX 20 kb) [file 13075_2015_843_MOESM1_ESM.docx]

**Additional file 1: Integrative Literature Review Search Strategy**

European Musculoskeletal Conditions Surveillance and Information Network (EUMUSC) Search Update (30)

**Medline:**

1 exp Arthritis/ (203995)

2 exp Arthritis, Rheumatoid/ (93390)

3 rheumatoid arthritis.tw. (74678)

4 RA.tw. (48413)

5 rheumatoid.tw. (85118)

6 rheumatic.tw. (30323)

7 exp Osteoarthritis/ (44111)

8 osteoarthritis.tw. (33600)

9 osteoarthrosis.tw. (2818)

10 Osteoarthritides.tw. (2)

11 Osteoarthroses.tw. (20)

12 Degenerative arthritis.tw. (973)

13 Degenerative Arthritides.tw. (10)

14 arthrosis.tw. (4447)

15 arthroses.tw. (436)

16 arthritides.tw. (1137)

17 arthritis.tw. (120642)

18 arthritic.tw. (7633)

19 Osteoarthrosis Deformans.tw. (128)

20 1 or 2 or 3 or 4 or 5 or 6 or 7 or 8 or 9 or 10 or 11 or 12 or 13 or 14 or 15 or 16 or 17 or 18 or 19 (279909)

21 exp Quality Indicators, Health Care/ (13450)

22 Quality indicator$.tw. (3829)

23 performance indicator$.tw. (1765)

24 process indicator$.tw. (445)

25 structure indicator$.tw. (27)

26 outcome$ indicator$.tw. (919)

27 quality parameter$.tw. (2380)

28 process parameter$.tw. (1795)

29 structure parameter$.tw. (334)

30 outcome$ parameter$.tw. (2618)

31 performance parameter$.tw. (1234)

32 21 or 22 or 23 or 24 or 25 or 26 or 27 or 28 or 29 or 30 or 31 (26097)

33 20 and 32 (287)

34 limit 33 to yr="2010 -Current" (141)

**EMBASE**:

Database: Embase <1974 to 2015 January 13>

Search Strategy:

1 rheumatoid arthritis.tw. (108764)

2 RA.tw. (76126)

3 rheumatoid.tw. (121960)

4 rheumatic.tw. (43078)

5 osteoarthritis.tw. (51365)

6 osteoarthrosis.tw. (3731)

7 Osteoarthritides.tw. (1)

8 Osteoarthroses.tw. (35)

9 Degenerative arthritis.tw. (1242)

10 Degenerative Arthritides.tw. (13)

11 arthrosis.tw. (6146)

12 arthroses.tw. (493)

13 arthritides.tw. (1540)

14 arthritis.tw. (174649)

15 arthritic.tw. (10382)

16 Osteoarthrosis Deformans.tw. (153)

17 Quality indicator$.tw. (6264)

18 performance indicator$.tw. (2667)

19 process indicator$.tw. (636)

20 structure indicator$.tw. (50)

21 outcome$ indicator$.tw. (1306)

22 quality parameter$.tw. (3873)

23 process parameter$.tw. (3360)

24 structure parameter$.tw. (562)

25 outcome$ parameter$.tw. (4328)

26 performance parameter$.tw. (1774)

27 1 or 2 or 3 or 4 or 5 or 6 or 7 or 8 or 9 or 10 or 11 or 12 or 13 or 14 or 15 or 16 (297349)

28 17 or 18 or 19 or 20 or 21 or 22 or 23 or 24 or 25 or 26 (24205)

29 27 and 28 (314)

30 limit 29 to yr="2010 -Current" (198)

**Websites searched:**

1. Canadian Institute for Health Information ([www.cihi.ca](http://www.cihi.ca))
2. The Arthritis Society ([www.arthritis.ca](http://www.arthritis.ca))
3. The Canadian Medical Association ([www.cma.ca](http://www.cma.ca))
4. The Arthritis Community Research and Evaluation Unit ([www.acreu.ca](http://www.acreu.ca))
5. The Canadian Arthritis Network ([www.arthritisnetwork.ca](http://www.arthritisnetwork.ca))
6. The Canadian Osteoarthritis Research Program ([www.osteoarthritisresearch.ca](http://www.osteoarthritisresearch.ca))
7. The Canadian Orthopaedic Association ([www.coa-aco.org](http://www.coa-aco.org))
8. The Canadian Rheumatology Association ([www.rheum.ca](http://www.rheum.ca))
9. Western Canada Waiting List Project ([www.wcwl.ca](http://www.wcwl.ca))
10. Ministry of Health and Long-Term Care, Ontario ([www.health.gov.on.ca](http://www.health.gov.on.ca))
11. The College of Physicians & Surgeons of Alberta ([www.cpsa.ab.ca](http://www.cpsa.ab.ca))
12. Fraser Institute ([www.fraserinstitute.org](http://www.fraserinstitute.org))
13. The Wait Time Alliance ([www.waittimealliance.ca](http://www.waittimealliance.ca))
14. The American College of Rheumatology ([www.rheumatology.org](http://www.rheumatology.org))
15. The National Quality Forum ([www.qualityforum.org](http://www.qualityforum.org))
16. Arthritis Foundation ([www.arthritis.org](http://www.arthritis.org))
17. American Arthritis Society ([www.americanarthritis.org](http://www.americanarthritis.org))
18. National Guidelines Clearinghouse ([www.guideline.gov](http://www.guideline.gov))
19. National Quality Measures Clearinghouse ([www.qualitymeasures.ahrq.gov](http://www.qualitymeasures.ahrq.gov))
20. The American Academy of Orthopaedic Surgeons ([www.aaos.org](http://www.aaos.org))
21. Arthritis Research UK ([www.arthritisresearchuk.org](http://www.arthritisresearchuk.org/arthritis-information/conditions/osteoarthritis.aspx))
22. The National Institute for Health and Care Excellence ([www.nice.org.uk](http://www.nice.org.uk))
23. Health & Social Care Information Centre, National Health Service Outcomes Framework ([www.hscic.gov.uk/iqi](http://www.hscic.gov.uk/iqi))
24. The King’s Fund ([www.kingsfund.org.uk](http://www.kingsfund.org.uk))
25. The European Musculoskeletal Conditions Surveillance and Information Network ([www.eumusc.net](http://www.eumusc.net))
26. The European Federation of National Associations of Orthopaedics and Traumatology ([www.efort.org](http://www.efort.org))
27. The European League Against Rheumatism ([www.eular.org](http://www.eular.org))
28. The Osteoarthritis Research Society International ([www.oarsi.org](http://www.oarsi.org))
29. The Bone and Joint Decade (bjdonline.org)
30. The Guidelines International Network ([www.g-i-n.net](http://www.g-i-n.net))
31. The Royal Australian College of General Practitioners ([www.racgp.org.au](http://www.racgp.org.au))
32. The Agency for Clinical Innovation Musculoskeletal Network ([www.aci.health.nsw.gov.au](http://www.aci.health.nsw.gov.au/networks/musculoskeletal))
33. The Australian Institute of Health and Welfare ([www.aihw.gov.au](http://www.aihw.gov.au))
